# Stress and Molecular Drivers for Cancer Progression: A Longstanding Hypothesis

**DOI:** 10.26717/bjstr.2021.37.005953

**Published:** 2021-07-12

**Authors:** J Wendel, A Verma, V Dhevan, SC Chauhan, MK Tripathi

**Affiliations:** 1 Department of Biology, College of Sciences, The University of Texas Rio GrandeValley, McAllen, TX 78539, USA; 2 South Texas Center of Excellence in Cancer Research, School of Medicine, Universityof Texas Rio Grande Valley, McAllen TX 78504, USA; 3 Department of Biomedical Engineering, Samrat Ashok Technological Institute, Vidisha,M.P., India; 4 Valley Baptist Hospital, Harlingen, TX 78550, USA; 5 Department of Surgery, School of Medicine, University of Texas Rio Grande Valley, Edinburg, TX 78501, USA; 6 Department of Immunology and Microbiology, School of Medicine, University of Texas Rio Grande Valley, McAllen, TX 78504, USA

**Keywords:** Stress, Cancer Recurrence, Drug Resistance, Cancer Progression, HPA Axis, HPT Axis

## Abstract

Stress management is becoming very important part of cancer patient care. Chronic stressors lead to boost tumorigenesis and promote cancer development, recurrence, and drug resistant leading to poor health outcomes. The Hypothalamic-Pituitary-Adrenal (HPA) axis, which is activated by stress, also regulates Hypothalamic-Pituitary-Thyroid (HPT) axis. Stress related changes in immune function and inflammatory response also leads to reduced immune surveillance resulting in tumorigenesis. This article explores the hormonal axis impacted by stress and how chronic stress can lead to poor outcome of a cancer patient.

## Introduction

Stress is a common factor in modern life. So much so, it is arguable that many have learned to ignore it rather than find ways to decrease it. The word brings to mind full schedules and anxiety riddled twilight hours, but what many don’t consider is the physiological response our body undergoes, especially under chronic stress. There is little argument against the idea that a diagnosis of cancer, at any stage, would increase a person’s daily baseline stress levels. This undoubtedly plays a role in many of the decisions and conscious thought made in the day-to-day activities of a newly diagnosed cancer patient, from activity level, to diet, to self-education and health literacy. Once again, little thought is given to the synergistic potential of cancer progression compounded with chronic stress. While the jury is out on the capability of stress to induce or initiate tumorigenesis, there is a consensus in the scientific community that chronic stress can create a physiological environment conducive to the increased proliferation, migration, invasion, drug resistance, and recurrence of existing cancer cells. The pathways and modulators involved are well described and models have been developed to uncover the multitude of factors involved in stress mediated cancer progression.

## Stress

Stress in this context exists in an acute phase as well as a chronic phase. Acute stress may be a useful evolutionary tool, altering the decision-making process in individuals [1]. However, chronic stress on the other hand, is a longer standing exposure to stress related hormones, and can cause an early wear and tear on peripheral organs and even psychosomatic degradation [2,3]. Factors that are most often linked to chronic stress range from socioeconomic factors and standing to physical illness. Health disparities have been linked to increased occurrence of chronic stress, tying the individual into a perpetual cycle of stress and inability to address the stressors, whether health related or economic [4,5]. Allostatic load, or the cumulative load and effect of chronic stress, has been directly correlated with heightened inflammatory responses, increased levels of oxidative stress, and DNA damage [4]. These outcomes are clear cut pro cancer metastasis factors and are widely accepted as influences in oncogenesis. Several studies have indicated significantly higher levels of allostatic load among minority groups and economically underprivileged peoples, which may be one facet of the health disparity and underlying prognoses of minority demographics [4–6].

Mechanisms of Stress It’s impossible to discuss stress without discourse on the hypothalamic-pituitary-adrenal axis (HPA) and the Sympathetic Nervous System (SNS). Whether the stress is psychological or physiological, the HPA axis is the primary route of influence. The release of the associated hormones in this axis alters a myriad of homeostatic functions. In an acute setting, this axis would initiate a response and subside once the stressor had been sufficiently removed or dealt with. However, it is not uncommon in today’s world for this axis to remain active, introducing a new health concern that spans a wide breadth of prevalent health concerns. Chronic stress has been shown to cause macroscopic changes in brain structure, dysregulation of the immune system, atherosclerosis, depression, poor sleep quality, and hypertension, among other disorders and chronic illnesses [7–10].

With the massive dysregulation of these systems, numerous tissues begin to elicit the appropriate microenvironment for cell proliferation, malignancy, and metastasis [11–13]. The HPA and SNS facilitate these changes through cell mediators like cytokines and hormones [14]. The cascade of signaling disruptions travels through a series of organs and metabolic processes which alter the normal physiological state in which the human body is intended to reside.

## Stress Hormones

**Figure 1:**
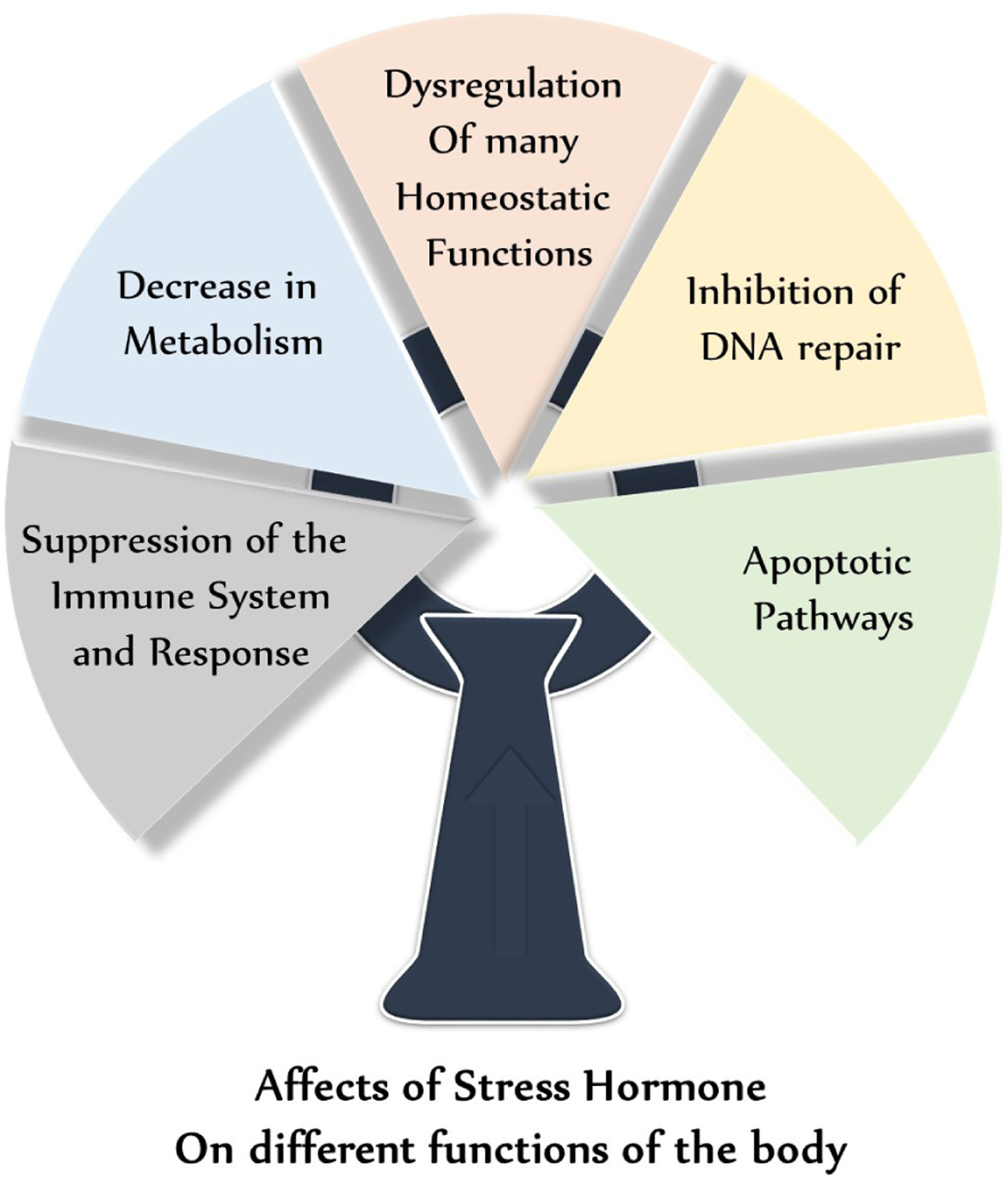
Schematic showing affects of stress hormones on different functions of body.

The physiological manifestations of stress start with the hypothalamic-pituitary-adrenal (HPA) axis. Stressors of various natures induce a release of Corticotropin Releasing Hormone (CRH) from the hypothalamus, which as its name suggests, stimulates the release of Adrenocorticotropic Hormone (ACTH) from the anterior pituitary. ACTH then stimulates the release of glucocorticoids, like cortisol, known as the stress hormone, from the adrenal cortex [15]. This class of hormone is known for modulating many systems in the body, including the suppression of the immune system and response, decrease in metabolism, dysregulation of many homeostatic functions, inhibition of DNA repair, and apoptotic pathways [2,3]. Figure 1 shows effects of stress hormones on different functions of body. Cortisol is a key player in stress, whether acute or chronic. Inversely related to our nocturnal hormone secretions, cortisol is normally suppressed at night due to a decrease in CRH release from the hypothalamus. In chronic stress, this decrease may happen later than intended, or may not happen at all.

Cortisol also plays a key role in driving insulin resistance, and as a result is a facet in diabetes mellitus and metabolic syndrome inset. Flattened diurnal cortisol rhythms, that is, chronic stress and elevated cortisol rhythms throughout the daytime, have been associated with poor prognosis and survival several types of cancer, including lung and breast cancer [16–18]. Those who suffer from chronic stress have also shown sustained increased levels of norepinephrine and epinephrine [19,20]. These neurotransmitters are known to play arole in angiogenesis and vascularization of tumors, a critical step in the growth and metastasis in cancer [21,22]. Norepinephrine has also been shown to increase cell migration in prostate cancer [23], increase proliferation, migration, and invasion of pancreatic cancer [24] and invasion of pancreatic cancer cells [25]. These neurotransmitters showcase the role of the stress regulated sympathetic nervous system in the progression of cancer in several tissue types, *in-vitro* and *in-vivo*.

**Figure 2:**
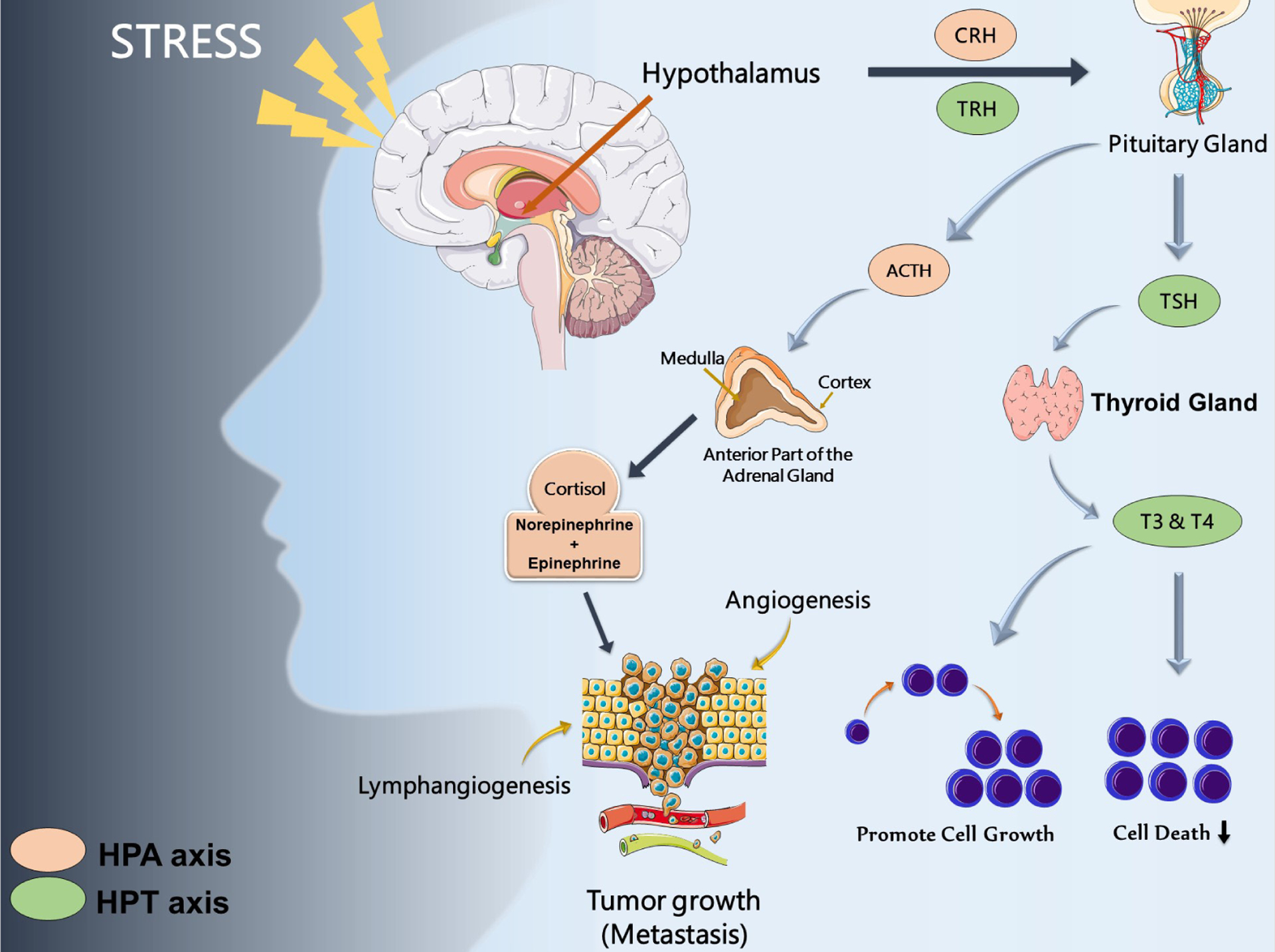
Stress hormones and cancer **Note:** Dysregulation of hypothalamic-pituitary-thyroid (HPT) and hypothalamic-pituitary-adrenal (HPA) due to socio-behavioral stress axis leads to cancer development and progression and impact processes such as apoptosis, angiogenesis, cell survival inhibition, lymphangiogenesis, tumor growth and metastasis.

The hypothalamic-pituitary-thyroid (HPT) axis has also been shown to communicate with the HPA axis [26] and the thyroid hormones T3 and T4 are able to regulate tumor cell cycle, growth, and death [27–29]. One study of 30,000 participants over a 9-year period found that those with primary subclinical hyperthyroidism had a higher risk of developing cancer, specifically of the lung and prostate tissues, while hypothyroidism had no significant relation to cancer risk [30]. Different cancer related mechanisms influenced predominantly by HPA, and HPT axis are summarized in (Figure 2).

## Stress and Inflammation

One component of chronic stress and increased cortisol levels is an amplified production of reactive oxygen species [4]. ROS and the oxidative stress they cause over an extended period can incite chronic inflammation potentially damaging cellular structure and cause mutations in otherwise healthy cells [31,32]. Oxidative stress can increase the levels of transcription factors (i.e. NF-κB, AP-1, p53, PPAR-γ, and β-catenin/Wnt) and upregulate genes associated with growth factors, inflammatory cytokines, chemokines, cell cycle regulatory molecules, and anti-inflammatory molecules [33]. It is likely that an individual suffering from chronic stress with a high allostatic load may have increased levels of these transcription factors and their associated genes.

These factors have also been used as biomarkers to identify chronic inflammation in patients with cervical, non-small cell lung, and colorectal cancers [34–36]. Chronic stress, and its eventual facilitation of chronic inflammation can create a highly suitable environment for mutations and cellular evolution to a proliferative state than otherwise observed. While inflammation is a normal immune mediated response to foreign infectious biota, chronic inflammation, like chronic stress, can cause premature deterioration in the systems it is affecting.

## Stress and Immunity

It is difficult to discuss the impact of inflammation without discourse on the immune system itself. Stress and stress hormones are well known to suppress the immune response and immune cells throughout the body. While one may normally think of the immune system in response to an infectious disease, the immune system plays a critical role in the detection and suppression of malignant cells. Several classes of immune cells, including tumor-infiltrating lymphocytes, cytotoxic T-cells, helper T-cells, and natural killer cells, have all been shown to play a role in elimination of tumor cells [37]. Therefore, inhibition of the immune response, as is seen in individuals suffering from chronic stress and high allostatic loads, can create an environment where the immune system is unable to maintain its vigilance in cellular maintenance, allowing for malignant cells to migrate, invade, and eventually proliferate into secondary tumor sites [38]. Paradoxically, an over-active immune system can play a paradoxical role in cancer progression and prognosis.

While chronic stress may inhibit the immune system, chronic inflammation, secondary to chronic stress, may cause an increased number of immune-cell infiltrates into tissues, promoting angiogenesis [39]. Angiogenesis is an imperative process in the development of tumor development and is correlated with poor prognosis [40]. The relationship of the immune system and cancer is contradictory at times, in part due to the wide breadth of responsibilities the immune system holds in maintaining homeostasis. With so many factors, classes of cells, transcription factors, and mechanisms of action, the immune system can both target cancer cells for destruction prior to metastasis, as well as promote the microenvironment needed for proliferative cell growth and tumorigenesis.

## Concluding Thoughts

Stress, specifically chronic stress, plays a far-reaching role in overall health, predisposition to disease, and progression of disease, well beyond the confines of cancer and the topics covered in this article. Even long term and potentially hereditary repercussions of gene expression and epigenetics are affected by the deregulation of the HPA axis. Current disease models, both *in-vitro* and *in-vivo*, can provide insight into this phenomenon and its interaction with general human health and advanced stage diseases. While society has deemed chronic stress to be the norm, and in some cases even applauded those who suffer from chronic stress as someone who just “stays busy”, the deleterious effect chronic stress has on homeostatic functions is much more intertwined with our well being than most will ascribe. Current and future research in chronic stress models should aim to elucidate the extent to which chronic stress, on a psychological, physiological, and molecular level, may be exacerbating these illnesses. While treating chronic stress may only be a sidecar to current medical treatments, this research could potentiate the necessary discussion on reducing levels of stress in a more meaningful round table of academia and healthcare.

## Abbreviations:

HPA: Hypothalamic-Pituitary-Adrenal
HPT: Hypothalamic-Pituitary-Thyroid
SNS: Sympathetic Nervous System
CRH: Corticotropin Releasing Hormone
ACTH: Adrenocorticotropic Hormone
